# Case Report: Moxibustion-induced burns leading to disseminated methicillin-resistant *Staphylococcus aureus* infection in a patient with type 2 diabetes mellitus

**DOI:** 10.3389/fendo.2026.1860646

**Published:** 2026-05-26

**Authors:** Hong Liang, Zhen-Fang Mao, Jin-Yu Huang, Yang Yang, Xue-Mei Li, Jie-Feng Mao, Qi Li, Yun-Bo Zhang, Wu-Xiao Wei

**Affiliations:** 1 The First Affiliated Hospital of Guangxi University of Science and Technology, Guangxi University of Science and Technology, Liuzhou, China; 2 Changde Hospital, Xiangya School of Medicine, Central South University (The First People’s Hospital of Changde City), Changde, China; 3 Youjiang Medical University for Nationalities, Baise, China

**Keywords:** burns, epidural abscess, hematogenous dissemination, methicillin-resistant *Staphylococcus aureus* (MRSA), moxibustion, peripheral neuropathy, type 2 diabetes, vertebral osteomyelitis

## Abstract

**Purpose:**

To assess methicillin-resistant *Staphylococcus aureus* (MRSA) bloodstream dissemination risk from moxibustion burns in type 2 diabetes and raise awareness of iatrogenic skin injuries in high-risk populations.

**Background:**

*Staphylococcus aureus* (*S. aureus*) causes bloodstream and metastatic infections, with a mortality rate up to 30%. In diabetic patients, minor skin injuries can lead to fatal systemic infections due to immune dysfunction and neuropathy. Moxibustion is widely used for chronic pain, but its thermal safety in high-risk groups is under-evaluated. Multiorgan MRSA dissemination involving the vertebrae, epidural space, chest wall, liver, lungs, and lymph nodes is extremely rare and challenging.

**Case presentation:**

We report the case of a 61-year-old male patient with type 2 diabetes mellitus and peripheral neuropathy. He developed second-degree burns with bleeding on the lower back following moxibustion therapy for back pain. The initial symptom was fever, followed by progressive bilateral lower limb weakness, urinary retention, and acute neurological deterioration. Laboratory tests revealed significantly elevated white blood cell count and inflammatory markers. Blood and wound cultures were both positive for MRSA. Thoracic spine MRI showed T8–T9 vertebral osteomyelitis and an epidural abscess with spinal cord compression. PET-CT further revealed a right chest wall abscess, a micro-abscess in liver segment S2, right upper lobe pneumonia, and mediastinal and hilar lymph node involvement. The patient was diagnosed with MRSA bloodstream dissemination secondary to moxibustion-induced burns. He received vancomycin for 6 weeks and local debridement but refused surgical drainage due to financial constraints. Although the infection was controlled, paraplegia and urinary retention persisted. The patient remains under follow-up.

**Conclusion:**

This case highlights that even minor burns caused by moxibustion in poorly controlled diabetic patients can serve as an occult portal for MRSA dissemination. Therefore, it is essential to routinely screen for peripheral neuropathy in diabetic patients before heat therapy and to perform strict skin monitoring afterward. For diabetic patients presenting with unexplained fever and neurological deficits, early blood and wound cultures as well as comprehensive imaging should be performed. Safety protocols for the application of traditional heat therapies in high-risk populations are urgently needed to address the current clinical safety gap.

## Introduction

*Staphylococcus aureus* (*S. aureus*) is a common opportunistic pathogen that colonizes the human skin and nasal mucosa (1). This bacterium can cause bloodstream infections with a mortality rate of up to 30%, and it may induce metastatic infections in more than one−third of affected patients, leading to conditions such as endocarditis, vertebral osteomyelitis, epidural abscess, psoas abscess, splenic abscess, and septic pulmonary embolism (2). Fulminant metastatic infection involving multiple non−contiguous organs remains rare and poses a major challenge to clinical diagnosis and treatment.

Diabetic patients often have neuropathy and microcirculatory disturbances, resulting in impaired heat sensation and delayed wound healing, which make them particularly susceptible to such infections (3, 4). Notably, even minor skin barrier damage (e.g., small burns, scalds, or abrasions) in diabetic patients can facilitate the invasion of methicillin-resistant *Staphylococcus aureus* (MRSA) through the compromised skin, progressing from localized infection to systemic dissemination due to immune dysfunction and a hyperglycemic environment that enhances bacterial virulence (5, 6).

Here we report the case of a 61−year−old male with type 2 diabetes mellitus who developed disseminated MRSA bloodstream infection following moxibustion−induced burns, with multiple metastatic foci including T8–T9 vertebral osteomyelitis, epidural abscess, right chest wall abscess, a micro−abscess in liver segment S2, right upper lobe pneumonia, and mediastinal and hilar lymph node infections. The patient initially presented with fever, followed by progressive bilateral lower limb weakness, urinary retention, and rapid neurological deterioration. This case underscores the critical importance of early microbiological diagnosis and personalized antimicrobial therapy. It also highlights the need for heightened awareness of skin injuries, especially in diabetic patients as a high−risk population. Although moxibustion has potential in chronic pain management (7, 8), its global application should be accompanied by rigorous safety monitoring, as this clinical safety gap is becoming increasingly evident.

## Case presentation

A 61-year-old male patient from the Guangxi Zhuang Autonomous Region was admitted with a 3-day history of fever and a 2-day history of bilateral lower limb weakness accompanied by urinary retention, following moxibustion−induced burns. He reported a 1−year history of chronic low back pain and had previously undergone moxibustion therapy for pain management at another hospital. He also had a 1−year history of type 2 diabetes mellitus, with poor blood glucose control in recent months. There was no history of implanted cardiac devices, prosthetics, central venous catheter placement, recent surgery, trauma, drug injection, hemodialysis, travel, respiratory disease, or urinary tract infection within the preceding 6 months.

On admission, physical examination revealed a temperature of 38.9 °C, pulse rate of 103 beats/min, blood pressure of 120/71 mmHg (1 mmHg = 0.133 kPa), and oxygen saturation of 99%. Lung auscultation disclosed coarse breath sounds with scattered wet rales. On the lower back, a 3 cm × 3 cm burn wound was observed along the midline, with local redness, swelling, bleeding, and elevated skin temperature (**Figure 1**). Neurological examination showed reduced light touch sensation in the bilateral T8–T12 dermatomes and loss of light touch sensation below the L1 level. Pinprick sensation was diminished in the bilateral T8–T12 dermatomes and absent below the L1 level, with decreased perianal sensation. Motor examination revealed muscle strength of 5/5 in both upper limbs. Lower limb strength was as follows: hip flexors (L2) 1/5, knee extensors (L3) 1/5, ankle dorsiflexors (L4) 0/5, extensor hallucis longus (L5) 0/5, and plantar flexors (S1) 0/5. Muscle tone in the lower limbs was decreased. Reflex examination showed reduced superficial abdominal reflexes; deep tendon reflexes were normal in the limbs. The cremasteric and anal reflexes were diminished.

**Figure 1:**
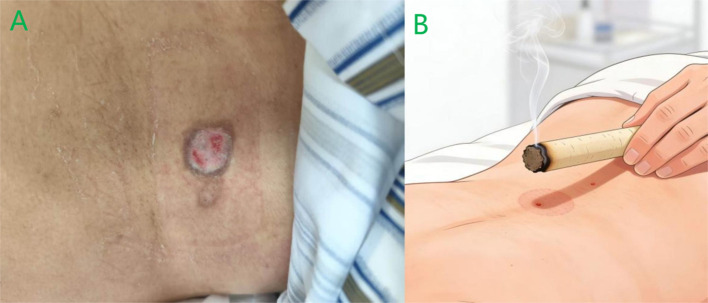
**(A)** Photograph of the patient’s moxibustion−induced burn on the lower back. **(B)** Schematic diagram illustrating moxibustion therapy for chronic pain management.

Laboratory results showed a white blood cell count of 23.02 × 10^9^/L (normal range: 3.5–9.5 × 10^9^/L), C−reactive protein of 147.9 mg/L (normal range: 0–10 mg/L), procalcitonin of 0.11 ng/mL (normal range: 0–0.05 ng/mL), and interleukin−6 of 704.90 pg/mL (normal range: 0–6.6 pg/mL). The erythrocyte sedimentation rate was 61 mm/h (normal range: 0–15 mm/h). Fasting plasma glucose was 9.3 mmol/L, and glycated hemoglobin (HbA1c) was 8.2%. Both pus and blood cultures were positive for MRSA (**Table 1**).

**Table 1:** Main laboratory test results at patient admission.

Variable	Result	Reference range
WBC (×10^9^/L)	23.02	3.5-9.5
CRP (mg/L)	147.9	0-10
PCT (ng/mL)	0.11	0-0.05
IL - 6 (pg/mL)	704.90	0-6.6
ESR (mm/h)	61	0-15
FPG (mmol/L)	9.3	3.9-6.1
HbA1c %	8.2	<6.5%
Blood culture	MRSA isolated	No growth
Wound secretion culture	MRSA isolated	No growth

WBC, white blood cell count; CRP, C-reactive protein; PCT, procalcitonin; IL-6, interleukin-6; ESR, erythrocyte sedimentation rate; FPG, fasting plasma glucose; HbA1c, glycated hemoglobin; MRSA, methicillin-resistant *Staphylococcus aureus*.

Transesophageal echocardiography showed normal findings. Plain and contrast−enhanced MRI of the thoracic spine showed patchy low signal on T1WI and high signal on T2WI in the T8–T9 vertebral bodies, with marked high signal on fat−saturated T2WI sequences, ill−defined margins, narrowing of the intervertebral disc spaces, and a patchy water−like signal at the posterior vertebral edge, consistent with vertebral osteomyelitis and epidural abscess with spinal cord compression; contrast−enhanced images demonstrated pronounced enhancement of the affected vertebral bodies and their posterior margins (**Figure 2**). PET−CT revealed increased FDG uptake in the mid−lower thoracic spine and posterior T8 vertebra, with SUVmax values of 8.46 and 5.84, respectively, as well as in the right 7th–8th intercostal space, liver segment S2, the posterior segment of the right upper lobe, the mediastinum, and bilateral hilar lymph nodes, with SUVmax values of 6.95, 5.72, 2.66, and 4.94, respectively (**Figure 3**).

**Figure 2:**
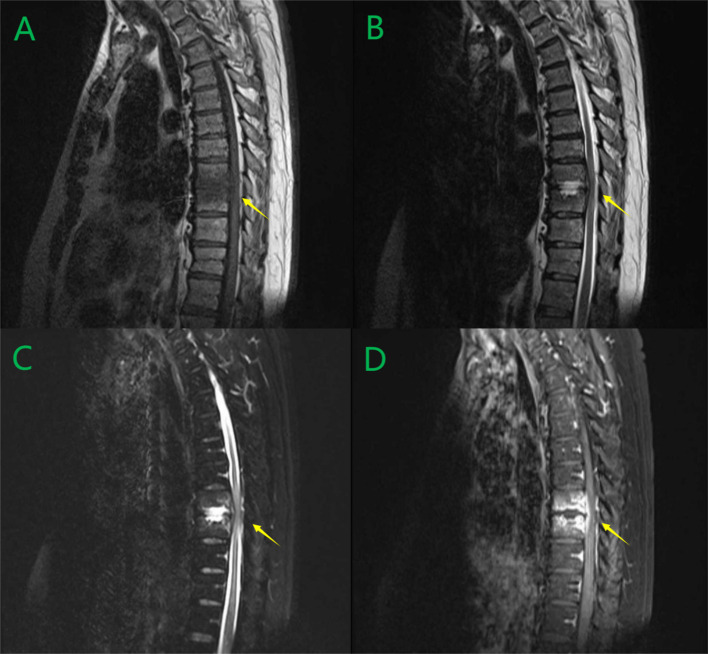
Plain and contrast−enhanced MRI of the thoracic spine showed patchy low signal on T1WI and high signal on T2WI in the T8–T9 vertebral bodies, with marked high signal on fat−saturated T2WI sequences, ill−defined margins, narrowing of the intervertebral disc spaces, and a patchy water−like signal at the posterior vertebral edge, consistent with vertebral osteomyelitis and epidural abscess with spinal cord compression; contrast−enhanced images demonstrated pronounced enhancement of the affected vertebral bodies and their posterior margins. **(A)** T1WI **(B)** T2WI **(C)** T2 STIR **(D)** Contrast-enhanced T1-weighted image.

**Figure 3:**
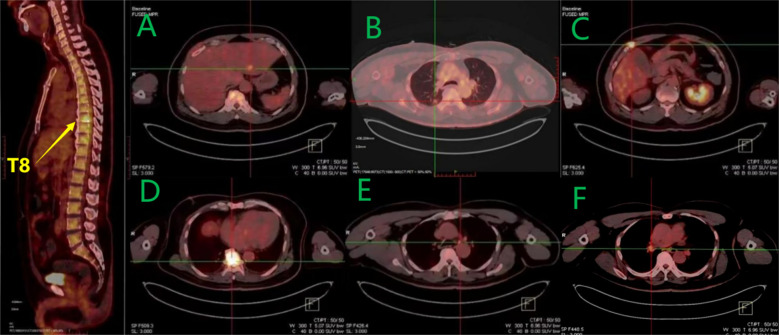
PET−CT imaging shows increased FDG uptake in the mid−to−lower thoracic spine and the posterior spinal cord at the T8 level (SUVmax 8.46 and 5.84, respectively). Increased FDG uptake is also observed in the right 7th–8th intercostal space, liver segment S2, the posterior segment of the right upper lobe, the mediastinum, and bilateral hilar lymph nodes, with SUVmax values of 6.95, 5.72, 2.66, and 4.94, respectively. **(A)** Lesion in liver segment S2; **(B)** Lesion in the posterior segment of the right upper lobe; **(C)** Lesion in the right 7th–8th intercostal space; **(D)** Lesion in the mid−to−lower thoracic spine and posterior spinal cord at T8; **(E, F)** Lesions in the mediastinum and bilateral hilar lymph nodes.

Because of its rare etiology, atypical symptoms, and multiorgan involvement, this case posed a diagnostic challenge. Based on the clinical history (burns and poorly controlled diabetes), positive MRSA cultures from multiple sites, and imaging findings, the final diagnosis was moxibustion−induced burns leading to MRSA bloodstream dissemination with multiple metastatic foci, including T8–T9 vertebral osteomyelitis, epidural abscess, right chest wall abscess, a micro−abscess in liver segment S2, right upper lobe pneumonia, and mediastinal and hilar lymph node involvement.

Based on the antimicrobial susceptibility testing results (**Table 2**), vancomycin was administered for a total of 6 weeks, during which therapeutic drug monitoring was performed and the target levels were achieved. After 4 weeks of treatment, blood cultures turned negative; after 6 weeks, inflammatory markers returned to normal, and imaging showed significant resolution of the primary and metastatic lesions, with no new lesions detected. Subsequently, the treatment was switched to cefazolin for further consolidation therapy. However, the patient’s bilateral lower limb paraplegia and urinary retention did not improve. He was then transferred to the Department of Rehabilitation Medicine for continued care and remains under follow-up.

**Table 2:** Patient’s susceptibility report.

Antibiotic	Susceptibility	MIC (μg/mL)
Ciprofloxacin	S	<=0.5
Tigecycline	S	<=0.12
Gentamicin	S	<=0.5
Levofloxacin	S	0.25
Moxifloxacin	S	<=0.25
Oxacillin	R	<=0.25
Penicillin	R	0.06
Quinupristin/Dalfopristin	S	<=0.25
Rifampin	S	<=0.5
Trimethoprim/Sulfamethoxazole	S	<=10
Linezolid	S	2
Vancomycin	S	>=32

S, Susceptible; R, Resistant; MIC, minimum inhibitory concentration.

The timeline illustrates the sequence of events from moxibustion-induced burn to admission, diagnosis, treatment response, and long-term outcomes. Key milestones include the onset of fever and neurological deficits, positive blood and wound cultures, initiation of vancomycin, PET-CT findings, culture conversion, and residual paraplegia (**Figure 4**).

**Figure 4:**
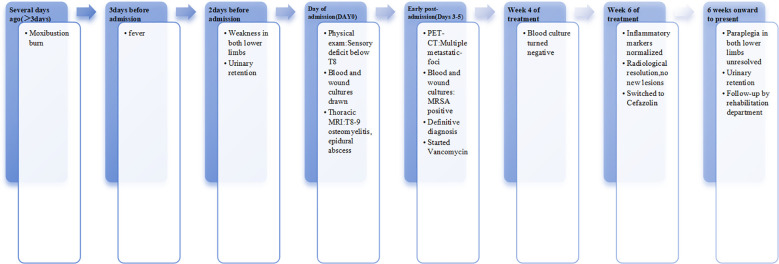
The timeline of the patient’s clinical course and treatment.

## Discussion

*S. aureus* is the leading pathogen responsible for pyogenic infections in humans (9). Approximately 20%–41% of the global population is transiently or persistently colonized by this Gram−positive bacterium (10). According to the Global Burden of Disease Study 2021, *S. aureus* accounted for approximately 325,000 deaths and 4.766 million disability−adjusted life years (DALYs) worldwide, with China alone contributing about 185,000 deaths, underscoring the substantial global and national burden of this pathogen (11). In individuals with comorbidities such as advanced age, diabetes, cardiovascular disease, renal impairment, or HIV infection, or in those with immunocompromised states such as neutrophil dysfunction, colonizing *S. aureus* can readily breach the skin barrier, enter the bloodstream, and cause severe systemic infections such as sepsis (12). The onset of *S. aureus* bacteremia is typically acute, with high fever observed in over 90% of patients, often accompanied by systemic toxic symptoms including chills, headache, and generalized myalgia (2). In the present case, hematogenous dissemination of MRSA secondary to moxibustion−induced burns resulted in multifocal infections involving vertebral osteomyelitis, epidural abscess, liver abscess, and pneumonia. Such a presentation is clinically rare and poses a significant challenge to both diagnosis and management. Although the presentation of this case is rare, other cases of moxibustion-related infections have been reported, such as pyogenic liver abscess caused by *Streptococcus intermedius*, and spinal epidural abscess caused by *Group B Streptococcus* (i.e., Streptococcus agalactiae), indicating that even local thermal injuries in high-risk patients can develop into life-threatening disseminated disease (13, 14).

The hyperglycemic environment not only impairs neutrophil chemotaxis, phagocytosis, and bactericidal activity but also facilitates colonization and biofilm formation of *S. aureus* on the skin surface (5, 15). Moreover, HbA1c levels in diabetic patients have been reported to correlate positively with the biofilm-forming capacity of *S. aureus* (15). In the present case, the patient’s HbA1c was 8.2%, reflecting poor glycemic control, which likely contributed to the rapid progression from a minor burn to invasive infection. The trauma disrupted the mechanical integrity of the skin barrier (6), permitting colonizing MRSA to enter the bloodstream. Coupled with virulence factors such as Panton–Valentine leukocidin and staphylokinase, the bacteria evaded host immune clearance (16, 17) and subsequently disseminated hematogenously to highly vascularized organs or those rich in reticuloendothelial tissue, including the bones and liver (18).

The patient’s clinical presentation was notably nonspecific. Fever was the initial symptom, followed by progressive bilateral lower limb weakness, urinary retention, and neurological deterioration, which could easily be misinterpreted as acute myelitis or isolated lumbar spine pathology. Thoracic spine MRI revealed T8–T9 vertebral osteomyelitis with an epidural abscess; however, limiting the assessment to the spine alone might have overlooked additional lesions in the liver, lungs, chest wall, and lymph nodes. Current literature recommends that all patients with *S. aureus* bacteremia undergo transthoracic echocardiography to screen for endocarditis, with high-risk patients requiring transesophageal echocardiography for definitive diagnosis (2). Advanced imaging modalities such as PET−CT or ^68^Ga−FAPI−04 PET/MR can further delineate the full extent of infection, providing essential guidance for targeted antimicrobial therapy (2, 19). Therefore, in diabetic patients presenting with unexplained fever and neurological deficits, particularly those with a recent history of skin trauma or heat therapy, a high index of suspicion for hematogenous dissemination of *S. aureus* is warranted. Early blood and wound cultures, together with comprehensive whole-body imaging, are crucial for timely diagnosis and management.

Currently, for patients at high risk of MRSA infection, antibiotics effective against MRSA, including vancomycin or daptomycin, should be initiated (2). For severe MRSA infections, AUC−guided vancomycin dosing is recommended, with a target AUC/MIC ratio (assuming an MIC of 1 mg/L) of 400–600 mg·h/L, and loading doses calculated based on actual body weight for both adults and children (20). The course of antibiotics varies according to disease complexity: for low−risk, uncomplicated cases, a 2−week course is often sufficient, whereas for high−risk, complicated cases, 4 to 6 weeks or even longer may be required, depending on the response to treatment (2). In the case of epidural abscess, the patient opted for conservative treatment and refused surgical drainage due to financial constraints. Although the infection was controlled, paraplegia did not recover. Alton et al. reported that diabetes, elevated CRP, leukocytosis, and positive blood cultures were significant predictors of medical treatment failure in spinal epidural abscess, and that delayed surgery after failed medical management resulted in significantly poorer motor recovery compared to early surgical intervention (21). The present case further illustrates a critical limitation of antibiotic-only therapy: even when microbiological eradication is achieved, irreversible neurological injury may have already occurred in the absence of timely surgical decompression. Indeed, Spernovasilis et al. demonstrated that in patients with MRSA infection or severe neurological impairment, conservative management carries an increased risk of failure (14). Moreover, it highlights a real-world challenge in resource-limited settings, where financial barriers may preclude patients from receiving guideline-recommended surgical care, thereby contributing to poor functional outcomes. Collectively, these observations reinforce that delayed treatment may lead to permanent neurological sequelae or even death (22).

## Conclusion

This case underscores the role of trauma, particularly iatrogenic thermal injury, as a potential trigger for hematogenous dissemination of MRSA, especially in high-risk populations such as patients with diabetes. Early recognition, prompt blood and wound cultures, comprehensive whole-body imaging, and individualized antimicrobial therapy are essential for reducing mortality and improving outcomes. Moreover, as traditional moxibustion therapy gains global popularity, strict procedural protocols and careful skin monitoring should be implemented for vulnerable populations, including diabetic patients, to prevent similar severe adverse events. Future studies should further investigate the link between traditional heat therapies and invasive infections and aim to establish more comprehensive diagnostic and therapeutic guidelines.

## Data Availability

The original contributions presented in the study are included in the article/supplementary material. Further inquiries can be directed to the corresponding authors.
